# Role of Matrix Metalloproteinases and Tissue Inhibitors in Sepsis Pathogenesis

**DOI:** 10.3390/medsci14030370

**Published:** 2026-07-03

**Authors:** Rasime Sevgi Cenan, Ayşegül Karaoğlan, Hatice Paşaoğlu

**Affiliations:** Department of Clinical Biochemistry, Gazi University Hospital, 06560 Ankara, Turkey

**Keywords:** sepsis, matrix metalloproteinases, tissue inhibitors of matrix metalloproteinases, inflammation, biomarkers

## Abstract

Background: This study investigates the role of MMPs and TIMPs in sepsis pathogenesis. We aim to assess multiple potential biomarkers including MMP-1, MMP-2, MMP-3, MMP-8, MMP-9, MMP-13, TIMP-1, TIMP-2, TIMP-3 in serum, brain and kidney tissue samples. To this date, there is not such extensive assessment of MMP and TIMP in sepsis pathogenesis. Methods: Experimental sepsis was generated using an LPS-induced endotoxemia model in rats. After 24 h, serum, brain and kidney samples were collected. Levels of MMP-1, MMP-2, MMP-3, MMP-8, MMP-9, MMP-13, and TIMP-1, TIMP-2, TIMP-3 were measured by ELISA. Inflammatory cytokines (TNF-α, IL-6, IL-10, and IFN-γ) were analyzed to assess the inflammatory response. Results: Analysis revealed that MMP-1 concentrations were elevated in kidney and brain tissues, as well as in serum in the sepsis group. MMP-2 levels exhibited statistically significant increases in brain tissues compared to controls. Elevations in MMP-3 were observed across brain, kidney and serum samples. MMP-8 levels were significantly increased in the serum. MMP-9 concentrations were higher in serum and kidney samples of septic rats compared with controls, while MMP-13 levels were elevated in kidney tissues of the sepsis group. TIMP measurements also revealed tissue-specific elevations in sepsis. TIMP-1 was significantly increased in kidney; TIMP-2 showed significant rises in brain tissue and serum; and TIMP-3 was markedly elevated in kidney tissues. Conclusions: A major strength of our study is the simultaneous assessment of multiple parameters. Our findings offer preliminary evidence of tissue-specific MMP and TIMP responses in sepsis and may guide future research exploring their biomarker potential. We anticipate that these results will inform both future clinical and pharmacological research and assist in the interpretation of subsequent studies.

## 1. Introduction

Sepsis is a complex clinical syndrome characterized by life-threatening organ dysfunction resulting from a dysregulated host response to infection and accompanied by severe physiological, pathological and biochemical changes. Diagnosis of sepsis relies on the patient’s medical history, physical examination, identification of infectious agents by blood culture, PCR and various laboratory assessments, including biomarkers, which can support accurate identification. Biomarkers in sepsis provide valuable information regarding diagnosis, treatment efficacy, and prognosis, motivating persistent investigation by researchers. However, because the pathophysiology of sepsis remains incompletely understood, no single biomarker can currently provide all the information required for diagnosis, prognosis, and clinical management [1,2,3,4].

Frequently investigated biomarkers comprise IL-6, CRP, procalcitonin, presepsin (soluble CD14 subtype, sCD14-ST), soluble urokinase plasminogen activator receptor (suPAR), and triggering receptor expressed on myeloid cells-1 (TREM-1). Studies evaluating these biomarkers have demonstrated promising results in septic patients. The investigation of sepsis biomarkers remains an active research area, with ongoing studies continuously enhancing the evidence base [2,3].

Timely identification and rapid intervention in sepsis are critical for patient survival. Differentiating septic patients from those with systemic inflammatory response syndrome (SIRS) triggered by non-infectious causes can be challenging. Early administration of antimicrobials is a cornerstone of sepsis management; however, careful stewardship is essential to prevent antibiotic resistance. Improving diagnostic accuracy can facilitate rational initiation and, when appropriate, deescalation of antimicrobial therapy and mitigating resistance risks [4,5,6,7].

A biomarker designed for early detection of hospital-acquired infections should be amenable to repeated measurements and provide predictive value for nosocomial infections and sepsis. When sepsis is suspected in a patient, a biomarker should deliver rapid results, demonstrate specificity for sepsis, and yield consistent findings across diverse patient populations. Furthermore, biomarkers intended to monitor therapeutic response should allow frequent measurements, facilitate assessment of treatment efficacy, detect early recurrence, and help determine optimal duration of antibiotic therapy [8,9].

Although biomarkers possess potential to achieve these goals, no single biomarker has been universally accepted as definitive for sepsis diagnosis. Ongoing investigations into sepsis biomarkers aim to deepen understanding of the condition and improve patient care. An ideal sepsis biomarker should allow early detection, enable prompt initiation of antibiotics, exhibit high specificity for distinguishing SIRS of infectious versus non-infectious origin, differentiate bacterial sepsis from other infections, provide prognostic insight, and guide antimicrobial therapy. Biomarkers can offer advantages at multiple stages of sepsis diagnosis, monitoring, and treatment [6,8,9].

The primary objective of this study was to elucidate the role of matrix metalloproteinases (MMPs) and tissue inhibitors of metalloproteinases (TIMPs) as potential biomarkers in sepsis pathogenesis. Specifically, the study assessed MMPs (MMP-1, MMP-2, MMP-3, MMP-8, MMP-9, MMP-13) and TIMPs (TIMP-1, TIMP-2, TIMP-3) in the context of sepsis. Alongside clinically relevant cytokines (TNF-α, IL-6, IL-10, IFN-γ), MMP and TIMP levels were measured in the brain and kidney tissues, as well as in serum. To our knowledge, no previous study has simultaneously evaluated such a comprehensive panel of MMP and TIMP enzymes. This novel approach is anticipated to make a significant contribution to the current literature.

## 2. Materials and Methods

This study received approval from the Local Ethics Committee for Animal Experiments at Gazi University Rectorate on 6 January 2023, with the approval number E-66332047, and was funded by the Scientific Research Projects of Gazi University under project code TDK-2023-8958.

The experimental sepsis model was established at the Gazi University Laboratory Animal Breeding and Experimental Research Center (GÜDAM). Subsequent experimental procedures were conducted at the Research Laboratory of the Department of Medical Biochemistry, Faculty of Medicine, Gazi University.

A sensitivity power analysis for two independent groups with *n* = 6 per group, two-sided α = 0.05, and 80% power indicated that the study could reliably detect only very large standardized differences (Cohen’s d approximately 1.80). Estimated power would be low for smaller effects (for example, approximately 24% for d = 0.80 and 47% for d = 1.20), so non-significant findings should not be interpreted as evidence of no biological effect.

The sample size was determined in accordance with the recommendations of the Institutional Animal Care and Use Ethics Committee, which required the use of the minimum number of animals necessary to achieve the study objectives in line with the principles of animal welfare. Accordingly, a total of 12 animals were allocated to the study, and no animals were excluded from the analysis. The animals were randomly assigned to the experimental groups.

Twelve 2-month-old female Wistar Albino rats weighing approximately 250 g were used in the study. Following administration, animals were maintained under a 12 h light/dark cycle and provided free access to standard chow and tap water.

Animals were randomly assigned to experimental groups and samples were coded with unique identifiers. Serum and tissue samples obtained from all 12 animals were labeled with randomly generated identification codes using Microsoft Excel. (Microsoft Excel for Microsoft 365, 2020) The code list linking sample identifiers to treatment groups was maintained separately by an independent investigator and was not accessible to the researchers performing the ELISA assays until all measurements had been completed.

The rats were divided into two groups, each containing six animals: the SHAM Group and the Sepsis Group.

1. Control (SHAM) Group: Rats in this group received 1 mL of physiological saline (PS) via intraperitoneal injection. Euthanasia was performed 24 h post-injection.
2. Sepsis Group: Rats in this group were administered 16 mg/kg of lipopolysaccharide (LPS, derived from *Escherichia coli*) intraperitoneally. Euthanasia was carried out 24 h later.

Following intraperitoneal injection, each animal was assigned a unique identification number, and all samples were coded to ensure blinding. Consequently, the investigators performing the ELISA analyses were unaware of the group allocation of the samples throughout the experimental and analytical procedures.

Twenty-four hours after administration, the animals’ general conditions, eye appearance, fur color, and activity levels were monitored to assess sepsis development.

At the end of the 24 h period, rats were euthanized under ketamine and xylazine anesthesia, and blood samples, as well as tissues from the brain and kidney were collected.

Blood samples were allowed to clot at room temperature for 20 min, then centrifuged at 3000 RPM for 20 min in serum tubes. The serum was separated using a pipette and stored at −80 °C until analysis.

Tissue samples were washed in physiological solution, flash-frozen in liquid nitrogen, and stored at −80 °C until further processing.

Tissues were removed from storage, and 100 mg portions were weighed. Using a Teflon-tipped homogenizer, tissues were homogenized in ice-cold PBS buffer (pH 7.4) at a ratio of 1:9 (tissue weight (g), PBS volume (mL)). The homogenates were centrifuged at 12,000 RPM for 15 min at 4 °C, and the supernatants were collected and stored at −80 °C until use.

ELISA assays were performed using BT-LAB kits (Bioassay Technology Laboratory, Shanghai, China) according to the manufacturer’s instructions. TNF-α, IL-6, IL-10, IFN-γ, MMP-1, MMP-2, MMP-3, MMP-8, MMP-9, MMP-13, TIMP-1, TIMP-2 and TIMP-3 concentrations were determined using a sandwich ELISA kit following the manufacturer’s protocol. The assay was based on a microplate pre-coated with a specific capture antibody. Standards and samples were added to the wells, followed by incubation with a biotinylated detection antibody and streptavidin-HRP conjugate. After incubation and washing steps to remove unbound material, chromogenic substrate solutions were added, resulting in a color reaction proportional to the amount of protein present in the samples. The reaction was stopped with an acidic stop solution, and optical density was measured at 450 nm using a microplate reader. Concentrations were calculated using a standard curve generated from known standards supplied with the kit. Absorbance readings from blank wells were subtracted from all samples and standards. The standard curve and its equation were generated, and sample concentrations were calculated accordingly.

Statistical analyses were carried out using SPSS version 25. Levels of proteins (TNF-α, IL-6, IL-10, IFN-γ, MMP-1, MMP-2, MMP-3, MMP-8, MMP-9, MMP-13, TIMP-1, TIMP-2, TIMP-3) in serum, brain and kidney tissues were compared between experimental and control groups. Independent-group tests were preferred because the Sham and LPS groups consisted of different animals. Shapiro–Wilk tests were used to assess normality. Normally distributed variables were compared using independent-samples *t*-tests, and non-normally distributed variables were compared using Mann–Whitney U tests. Benjamini–Hochberg false discovery rate (FDR) correction was applied across the 39 article-scope marker-by-compartment comparisons; q < 0.05 was considered statistically significant. Results are reported with raw *p*-values, BH-FDR q-values, and standard errors.

Benjamini–Hochberg FDR correction was used because multiple markers were tested across multiple compartments. This method limits the expected proportion of false-positive findings among the comparisons declared significant while being less conservative than Bonferroni correction, which is appropriate for this exploratory biomarker panel.

## 3. Results

The primary objective of this study was to investigate the role of matrix metalloproteinases (MMPs) and their tissue inhibitors (TIMPs) in sepsis. While the systemic inflammatory response triggered during sepsis is recognized as a key factor in organ failure, the specific contribution of the MMP/TIMP axis to this pathological process is not fully understood.

We hypothesized that sepsis would provoke a widespread inflammatory response characterized by elevated MMP levels and a concomitantly insufficient TIMP response. To address this, we analyzed brain and kidney tissues, along with serum samples, to evaluate organ involvement in sepsis. By carrying out a comparative assessment of MMP-1, MMP-2, MMP-3, MMP-8, MMP-9, MMP-13 and TIMP-1, TIMP-2, TIMP-3 across these biological specimens, we aimed to provide evidence highlighting the pathophysiological relevance of MMP/TIMP imbalance in sepsis. Additionally, we evaluated inflammatory mediators including TNF-α, IL-6, IL-10 and IFN-γ. MMP-1, MMP-2, MMP-3, MMP-8, MMP-9, MMP-13, TIMP-1, TIMP-2, TIMP-3, TNF-α, IL-6, IL-10, and IFN-γ levels were compared between Sham and LPS groups using independent-group statistics and FDR correction. (Table 1).

**Figure 1:**
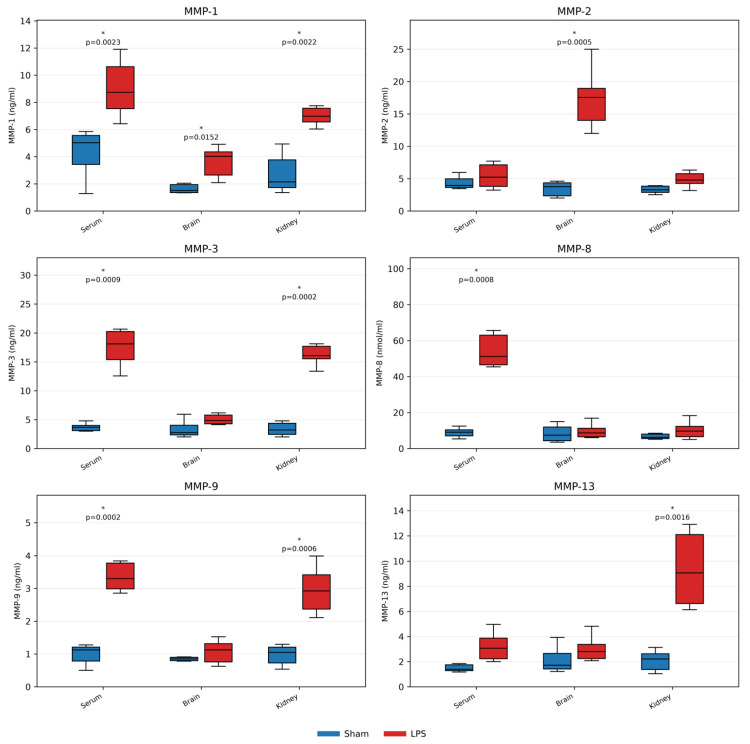
Box plots show serum, brain, and kidney concentrations of MMP-1, MMP-2, MMP-3, MMP-8, MMP-9, and MMP-13. Blue boxes indicate Sham animals (*n* = 6), and red boxes indicate LPS animals (*n* = 6). Asterisks indicate comparisons significant after Benjamini–Hochberg FDR correction (q < 0.05). Exact *p*-values for FDR-significant comparisons: MMP-1: serum *p* = 0.0023, brain *p* = 0.0152, kidney *p* = 0.0022; MMP-2: brain *p* = 0.0005; MMP-3: serum *p* = 0.0009, kidney *p* = 0.0002; MMP-8: serum *p* = 0.0008; MMP-9: serum *p* = 0.0002, kidney *p* = 0.0006; MMP-13: kidney *p* = 0.0016.

Analysis revealed that MMP-1 levels were higher in serum samples. (Sham 4.305 ± 0.741 vs. LPS 9.032 ± 0.884; *p* = 0.0023, q = 0.0056), brain tissue samples (Sham 1.950 ± 0.417 vs. LPS 3.615 ± 0.478; *p* = 0.0152, q = 0.0328), and kidney tissue samples. (Sham 2.743 ± 0.611 vs. LPS 7.680 ± 0.879; *p* = 0.0022, q = 0.0056). MMP-2 levels were higher in brain tissue samples (Sham 3.426 ± 0.484 vs. LPS 17.389 ± 1.920; *p* = 0.0005, q = 0.0034). MMP-3 levels were higher in serum samples (Sham 3.689 ± 0.282 vs. LPS 18.700 ± 2.202; *p* = 0.0009, q = 0.0041) and kidney tissue samples. (Sham 3.361 ± 0.480 vs. LPS 17.351 ± 1.652; *p* = 0.0002, q = 0.0030). MMP-8 levels were found higher in serum samples (Sham 8.859 ± 1.074 vs. LPS 58.295 ± 7.063; *p* = 0.0008, q = 0.0039). MMP-9 levels were higher in serum samples (Sham 0.990 ± 0.130 vs. LPS 3.537 ± 0.322; *p* = 0.0002, q = 0.0030) and kidney tissue samples (Sham 0.969 ± 0.128 vs. LPS 2.953 ± 0.300; *p* = 0.0006, q = 0.0034). MMP-13 levels were higher in kidney tissue samples. (Sham 2.081 ± 0.344 vs. LPS 9.353 ± 1.254; *p* = 0.0016, q = 0.0056). (Figure 1).

**Figure 2:**
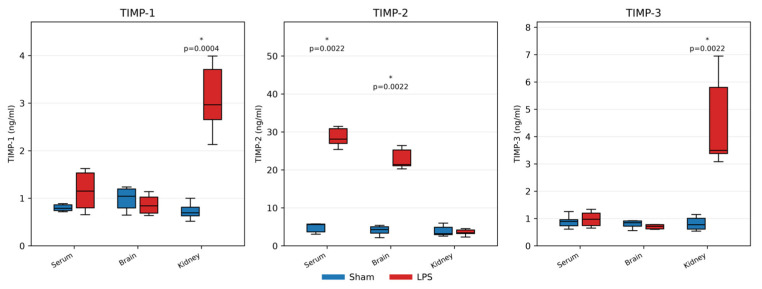
Box plots show serum, brain, and kidney concentrations of TIMP-1, TIMP-2, and TIMP-3. Blue boxes indicate Sham animals (*n* = 6), and red boxes indicate LPS animals (*n* = 6). Asterisks indicate comparisons significant after Benjamini–Hochberg FDR correction (q < 0.05). Exact *p*-values for FDR-significant comparisons: TIMP-1: kidney *p* = 0.0004; TIMP-2: serum *p* = 0.0022, brain *p* = 0.0022; TIMP-3: kidney *p* = 0.0022.

**Figure 3:**
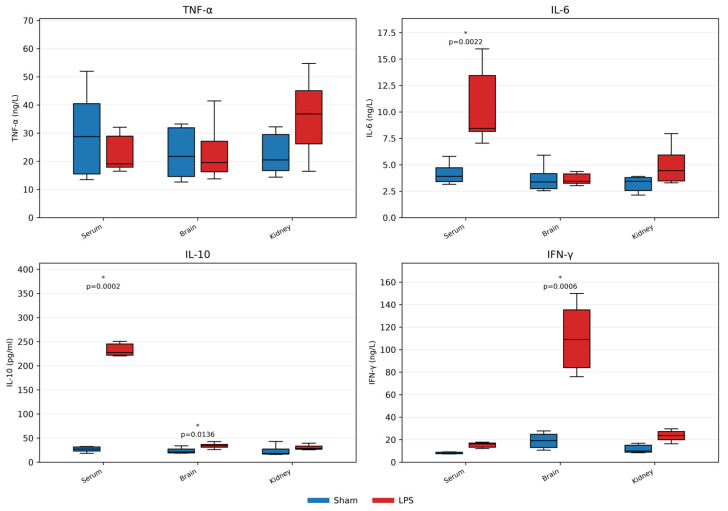
Box plots show serum, brain, and kidney concentrations of TNF-α, IL-6, IL-10, and IFN-γ. Blue boxes indicate Sham animals (*n* = 6), and red boxes indicate LPS animals (*n* = 6). Asterisks indicate comparisons significant after Benjamini–Hochberg FDR correction (q < 0.05). Exact *p*-values for FDR-significant comparisons: IL-6: serum *p* = 0.0022; IL-10: serum *p* = 0.0002, brain *p* = 0.0136; IFN-γ: brain *p* = 0.0006.

TIMP and cytokine measurements also showed compartment-specific changes. TIMP-1 levels were higher in kidney tissue samples, (Sham 0.728 ± 0.070 vs. LPS 3.092 ± 0.300; *p* = 0.0004, q = 0.0034). TIMP-2 levels were higher in serum samples (Sham 4.816 ± 0.557 vs. LPS 31.654 ± 3.762; *p* = 0.0022, q = 0.0056) and brain tissue samples. (Sham 4.073 ± 0.512 vs. LPS 25.086 ± 3.101; *p* = 0.0022, q = 0.0056). TIMP-3 was higher in kidney tissue samples. (Sham 0.813 ± 0.103 vs. LPS 4.487 ± 0.721; *p* = 0.0022, q = 0.0056). Regarding inflammatory cytokines, TNF-α showed no FDR-significant difference in serum, brain, or kidney. IL-6 levels were higher in serum samples, (Sham 4.162 ± 0.418 vs. LPS 10.511 ± 1.596; *p* = 0.0022, q = 0.0056). IL-10 levels were higher in serum samples (Sham 26.672 ± 2.385 vs. LPS 242.673 ± 23.373; *p* = 0.0002, q = 0.0030) and brain tissue samples. (Sham 23.810 ± 2.547 vs. LPS 34.207 ± 2.366; *p* = 0.0136, q = 0.0313). IFN-γ was higher in brain tissue samples. (Sham 19.029 ± 3.000 vs. LPS 110.616 ± 12.818; *p* = 0.0006, q = 0.0034). (Figure 2 and Figure 3).

## 4. Discussion

The present study demonstrated compartment-specific alterations in MMPs, TIMPs, and inflammatory cytokines in an LPS-induced experimental sepsis model. These findings suggest that extracellular matrix remodeling and inflammatory responses may differ between tissues during sepsis.

Cytokine analysis revealed significant increases in serum IL-6 and IL-10 concentrations, accompanied by elevated brain IL-10 levels. These findings are consistent with the simultaneous activation of pro-inflammatory and anti-inflammatory responses during sepsis [10]. Although TNF-α did not remain significant after FDR correction, elevated serum IL-6, increased IL-10 in serum and brain tissue, and enhanced cerebral IFN-γ concentrations collectively indicate substantial immune activation in this experimental model [11,12].

In a systematic review of patients with severe COVID-19, the nonsurvivor group exhibited persistently elevated IL-6 levels after treatment compared with survivors, suggesting that serial IL-6 measurements may be useful for monitoring treatment response and predicting prognosis. Although this systematic review evaluated patients with severe COVID-19 rather than sepsis, it provides valuable evidence regarding the prognostic significance of dynamic cytokine changes during systemic inflammatory conditions. The authors reported that reduced IL-6 and TNF-α levels after sepsis treatment in the intensive care unit, may be indicators of better prognosis and survival of patients with sepsis [13].

Similarly, IL-10 has attracted increasing interest as a clinically relevant biomarker. In current clinical practice, IL-10 is treated as a time-sensitive severity biomarker, particularly when assessed serially and interpreted as part of a biomarker panel in conjunction with organ dysfunction scores and markers of immune function, rather than as a stand-alone diagnostic marker [14]. Our observations are consistent with the literature but given the limited scale of our study, these findings should be considered hypothesis-generating rather than definitive.

The use of a single sampling time point represents an important limitation of the present study. Cytokines exhibit rapid and dynamic temporal changes during sepsis, with distinct release kinetics and relatively short half-lives. Consequently, measurements obtained at a single experimental time point may not fully capture the peak or temporal profile of individual cytokines. This limitation may partially explain the lack of significant changes observed for certain cytokines, particularly TNF-α, while also influencing the interpretation of the remaining cytokine measurements. Future studies incorporating serial sampling at multiple time points would provide a more comprehensive characterization of cytokine dynamics during experimental sepsis. In addition to the limitations associated with cytokine kinetics, the experimental model itself should also be considered when interpreting the present findings. Experimental murine models have substantially contributed to the current understanding of sepsis pathophysiology; however, their ability to fully represent the complexity of human sepsis remains a matter of ongoing debate [15,16].

Another limitation of the present study is the use of an LPS-induced endotoxemia model, which represents a simplified, noninfectious model of sepsis. Although LPS administration induces a robust systemic inflammatory response, it does not fully reproduce the complex pathophysiological features of clinical sepsis, including persistent infection, pathogen–host interactions, and the hyperdynamic cardiovascular phase commonly observed in human disease. Nevertheless, the LPS model was selected because it is a well-established, highly reproducible, and standardized experimental approach for investigating inflammatory mechanisms. Furthermore, the LPS protocol was standardized and routinely implemented in our laboratory, thereby ensuring methodological consistency and minimizing procedural variability throughout the study. Future studies using more clinically relevant polymicrobial sepsis models, such as cecal ligation and puncture, would help to validate and extend the present findings [17]. Despite these limitations, the present model provides a useful foundation for future mechanistic and translational investigations.

Matrix metalloproteinases (MMPs) have emerged as clinically important biomarkers owing to their central role in extracellular matrix (ECM) remodeling and the regulation of inflammatory processes. Increasing evidence has demonstrated their diagnostic, prognostic, and potential therapeutic value across a wide range of pathological conditions, including cardiovascular diseases, cancer, bone disorders, neurodegenerative diseases, and sepsis. Consequently, growing interest has focused on elucidating the contribution of individual MMPs to disease pathogenesis and evaluating their utility as biomarkers for disease severity, prognosis, and therapeutic monitoring [18].

The growing interest in MMPs as diagnostic and prognostic biomarkers in sepsis stems not only from their altered circulating levels during disease but also from their fundamental roles in regulating inflammatory responses. Beyond their classical function in extracellular matrix degradation, MMPs modulate inflammation by processing cytokines, chemokines, cell surface receptors, and other bioactive mediators, thereby influencing leukocyte recruitment, endothelial barrier integrity, and tissue remodeling. However, the biological functions of MMPs are highly context-dependent, with individual family members exerting both protective and detrimental effects depending on the stage and severity of inflammation. While certain MMPs contribute to the resolution of inflammation and tissue repair, excessive or dysregulated MMP activity may promote uncontrolled extracellular matrix degradation, vascular permeability, fibrosis, and organ dysfunction. This dual role highlights the complexity of interpreting MMP alterations in sepsis and underscores the need to evaluate individual MMPs within specific pathological and tissue contexts [19].

Dal-Pizzol et al. reported that MMP-2 protein levels and enzymatic activity may not parallel each other in the brains of septic animals, suggesting that MMP-2 activity is tightly regulated by endogenous inhibitors rather than being solely determined by protein expression [20]. In the present study, both MMP-2 and TIMP-2 protein levels were significantly increased in brain tissue following LPS-induced sepsis. These findings may reflect a compensatory response in which increased TIMP-2 expression is induced to counterbalance the elevation of MMP-2. Nevertheless, the persistence of elevated MMP-2 protein levels despite increased TIMP-2 suggests that the endogenous inhibitory response may be insufficient to completely restore protease–antiprotease homeostasis under septic conditions. Since only protein concentrations were evaluated in our study, it remains possible that MMP-2 enzymatic activity differed from its expression level, as previously proposed by Dal-Pizzol et al. Therefore, future studies integrating protein expression, enzymatic activity, and TIMP functional capacity are warranted to better elucidate the regulation of the MMP/TIMP axis during sepsis.

Previous experimental studies have suggested that global inhibition of MMPs may not be an appropriate therapeutic strategy because individual MMP have distinct and sometimes opposing biological functions during inflammation. Instead, selective targeting of specific MMPs, particularly MMP-8, has been proposed as a more promising therapeutic approach. Consistent with these observations, our study demonstrated significantly elevated serum MMP-8 levels in septic rats, accompanied by increased circulating IL-6 and IL-10 concentrations. Although causal relationships cannot be established from the present findings, the concurrent elevation of MMP-8 with key inflammatory cytokines supports the concept that MMP-8 is involved in the systemic inflammatory response during sepsis. Therefore, our results add to the growing body of evidence implicating MMP-8 in sepsis pathophysiology and warrants investigation in future mechanistic studies [21].

Alekhmimi et al. demonstrated that MMP-9 levels increased rapidly following the induction of sepsis in a murine fecal intraperitoneal model, with significant elevations detected in both blood and bronchoalveolar lavage fluid as early as 1 h after challenge. In the present study, serum MMP-9 concentrations were also significantly elevated in septic rats, supporting previous evidence that MMP-9 is upregulated during the systemic inflammatory response associated with sepsis. Unlike the serial sampling approach used by Alekhmimi et al., our study evaluated MMP-9 at a single time point (24 h after LPS administration), precluding assessment of its temporal expression profile. The persistence of elevated serum MMP-9 at this later stage suggests that MMP-9 remains involved in the inflammatory response [22].

Recent studies in patients with COVID-19 have also highlighted the involvement of the MMP/TIMP system in severe systemic inflammatory conditions. However, these studies have emphasized several important limitations, including heterogeneous sampling time points, the lack of standardized reporting of MMP expression, and the frequent omission of TIMP measurements, all of which complicate the interpretation of MMP activation and its clinical significance. Although the findings obtained in COVID-19 cannot be directly extrapolated to sepsis because of differences in disease pathophysiology, they underscore the importance of evaluating both MMPs and their endogenous inhibitors when investigating dysregulated inflammatory responses [23]. In our study, serum levels of MMP-1, MMP-3, MMP-8, MMP-9, MMP-13, and TIMP-2 were significantly elevated in septic rats. The simultaneous assessment of multiple MMPs together with TIMPs provides a more comprehensive characterization of protease–antiprotease imbalance during experimental sepsis and supports the concept that activation of the MMP/TIMP axis represents a consistent feature of systemic inflammation. Nevertheless, further studies incorporating serial sampling, functional enzyme activity, and clinical validation are required to clarify the temporal dynamics and biological significance of these alterations in sepsis.

Li et al. demonstrated that plasma TIMP-2 levels increased as early as 6 h after cecal ligation and puncture and continued to rise over time, preceding the development of biochemical evidence of acute kidney injury. Based on these findings, the authors proposed plasma TIMP-2 as a potential early biomarker of sepsis-associated acute kidney injury. Consistent with their observations, we also found significantly elevated serum TIMP-2 levels following LPS-induced sepsis. However, unlike their study, TIMP-2 expression was not significantly altered in kidney tissue. This discrepancy may be attributable to differences in experimental models, sampling strategies or tissue-specific regulation [24]. Importantly, our study was designed to comprehensively evaluate MMPs, TIMPs, and inflammatory cytokines at a single experimental time point rather than to characterize the temporal progression of kidney injury. Because renal function parameters and histopathological changes were not assessed, our findings should not be interpreted as evidence of sepsis-associated acute kidney injury. Instead, the observed increase in circulating TIMP-2 likely reflects the systemic inflammatory response to sepsis.

Serrano-Gomez et al. evaluated the prognostic value of circulating MMP-2, MMP-9, TIMP-1, TIMP-2, and their corresponding ratios in a cohort of patients with sepsis. Although TIMP-1 demonstrated relatively higher sensitivity and specificity than the other biomarkers, none of the evaluated markers showed sufficient predictive value for mortality. The differences between their findings and the present study should be interpreted in light of the distinct study objectives and experimental designs [25]. While their investigation focused on the prognostic performance of a limited number of circulating biomarkers in human sepsis, our study aimed to characterize tissue-specific alterations of the MMP/TIMP axis in an experimental LPS-induced sepsis model. Accordingly, we identified significant increases in brain MMP-2, serum MMP-9, kidney TIMP-1, and serum and brain TIMP-2. These observations suggest that alterations in the MMP/TIMP system may be compartment-specific and therefore may not be fully reflected by circulating biomarkers alone.

The biological relevance of the MMP/TIMP axis in sepsis is further supported by several prospective clinical cohort studies. Lorente et al. demonstrated that persistently elevated TIMP-1/MMP-9 ratios during the first week of severe sepsis were associated with increased mortality. A major strength of their study was the inclusion of a large patient cohort and serial measurements of TIMP-1, MMP-9, TNF-α, IL-10, and PAI-1. However, the authors acknowledged that only a limited number of MMPs and TIMPs were evaluated, restricting a comprehensive assessment of protease–antiprotease regulation [26]. Similarly, Forsblom et al. reported significantly increased plasma MMP-8 concentrations and MMP-8/TIMP-1 molar ratios in patients with severe *Staphylococcus aureus* bacteremia, while also emphasizing that differences in patient characteristics, disease severity, sampling time points, and biomarker measurement strategies complicate direct comparisons among studies [27].

Bojic et al. reported that lower circulating MMP-9 levels, higher TIMP-1 concentrations, and a reduced MMP-9/TIMP-1 ratio were associated with poorer survival in patients with sepsis following major abdominal surgery. Furthermore, these biomarkers correlated with indices of systemic inflammation, kidney and liver injury, coagulation abnormalities, metabolic disturbances, and disease severity, further supporting the clinical relevance of MMP/TIMP dysregulation during sepsis [28]. Although our study was not designed to evaluate survival or clinical outcomes, the observed alterations in serum MMP-9 and tissue-specific TIMP expression are consistent with the concept that disruption of the MMP/TIMP axis accompanies the systemic inflammatory response in sepsis.

A recent study by Tiwary et al. identified TIMP-1 as a promising candidate biomarker for the diagnosis of bloodstream infections and emphasized the need for larger studies comparing its diagnostic performance with established sepsis biomarkers such as procalcitonin. These findings further support the growing interest in TIMP-1 as a clinically relevant biomarker in systemic infections. Consistent with this concept, we observed significantly increased TIMP-1 expression in the kidney tissue of septic rats. Although our study was not designed to evaluate the diagnostic performance of TIMP-1 or to assess renal injury, the observed tissue-specific increase suggests that TIMP-1 participates in the local inflammatory response during experimental sepsis. These findings provide additional evidence supporting the biological involvement of TIMP-1 in sepsis and warrant further investigation of its tissue-specific role and potential biomarker value [29].

Jones et al. reported that elevated plasma MMP-3 and TIMP-1 levels were associated with organ dysfunction and mortality in patients with sepsis, highlighting the potential clinical significance of these biomarkers. In agreement with these findings, our study demonstrated significantly increased MMP-3 levels in both serum and kidney tissue, together with increased TIMP-1 expression in kidney tissue following LPS-induced sepsis. Although our experimental model was not designed to evaluate organ dysfunction or clinical outcomes, the observed alterations in MMP-3 and TIMP-1 support the concept that activation of the MMP/TIMP axis is an integral component of the septic inflammatory response [30].

Although our study was performed in an experimental LPS-induced sepsis model and was not designed to evaluate prognosis or mortality, our findings are consistent with the concept that dysregulation of the MMP/TIMP axis is a characteristic feature of sepsis. We observed significant alterations in several MMPs and TIMPs, including increased serum MMP-8, MMP-9, and TIMP-2, together with tissue-specific changes in MMP-2, TIMP-1, and TIMP-2. The concordance between our experimental findings and observations from independent clinical cohorts supports the biological relevance of MMP/TIMP dysregulation during sepsis. Furthermore, unlike the clinical studies that focused on a limited number of circulating biomarkers, our study simultaneously evaluated a broader panel of MMPs and TIMPs in serum and multiple organs, providing a more comprehensive characterization of tissue-specific protease–antiprotease regulation during experimental sepsis. This integrated approach may contribute to the identification of tissue-specific biomarker patterns and guide future translational studies.

This study has some limitations that should be considered when interpreting the findings. First, the relatively small sample size may limit the generalizability of the results. Second, all measurements were performed at a single experimental time point. Given the rapid and dynamic kinetics of cytokines during sepsis, single-point measurements may not fully capture their temporal expression patterns or peak concentrations. In addition, the LPS-induced endotoxemia model, although well established and highly reproducible for investigating inflammatory mechanisms, does not fully recapitulate the complex pathophysiology of human sepsis. Furthermore, only protein expression levels were evaluated, whereas histopathological assessment, gene expression analyses, and functional studies were not performed. Therefore, the present findings should be interpreted as preliminary observations that warrant further validation in larger studies incorporating serial sampling, complementary molecular and histopathological analyses, functional investigations, and more clinically relevant experimental sepsis models.

The present study offers several novel aspects. Unlike many previous studies that focused on a limited number of circulating biomarkers, we simultaneously evaluated a broad panel of matrix metalloproteinases (MMP-1, MMP-2, MMP-3, MMP-8, MMP-9, and MMP-13), their endogenous inhibitors (TIMP-1, TIMP-2, and TIMP-3), and key inflammatory cytokines (TNF-α, IL-6, IL-10, and IFN-γ) within the same experimental model. Second, biomarker expression was assessed not only in serum but also in brain and kidney tissues, allowing direct comparison between systemic and tissue-specific responses. This tissue-oriented approach provides additional insight into the compartment-specific regulation of extracellular matrix remodeling and inflammation during experimental sepsis. Our findings provide a comprehensive dataset that may serve as a valuable foundation for future mechanistic investigations and biomarker-oriented research in sepsis.

Collectively, these findings provide preliminary evidence of tissue-specific alterations in MMPs, TIMPs, and cytokines during experimental endotoxemia and may serve as a basis for future studies investigating their biological significance in sepsis.

## 5. Conclusions

Few studies have examined the potential roles of MMPs and TIMPs in sepsis. Although methodological differences existed among the experimental models, their findings collectively support several common conclusions. First of all, we must note that MMP or TIMP activity alone may not be solely beneficial or detrimental, as their effects depend on the overall protease environment. Therefore, the balance between MMPs and TIMPs determines their functional roles in sepsis. Our study was designed to simultaneously evaluate multiple enzymes, enabling us observe changes in MMP and TIMP levels.

Differences in experimental models and patient populations in previous studies is a limiting factor. While the LPS model successfully reproduces many inflammatory features of sepsis, it does not fully capture the complexity of the host response to a live infection and therefore represents only a partial model of the clinical syndrome. Therefore, the findings of the present study should be interpreted within the context of the experimental model used, and extrapolation to human sepsis should be made with caution.

Moreover, many prior studies did not assess multiple MMP and TIMP types simultaneously. In addition, samples were collected from kidney and brain tissues, enabling tissue-specific comparisons with the control group. Serum samples were also obtained, allowing comparisons between tissue and serum levels. We are aware that clinical studies are needed to confirm these findings. We believe our results will guide future sepsis biomarker research, especially through sequential sampling in patients at different stages of inflammation in sepsis and septic shock.

In conclusion, our findings suggest that tissue-specific alterations in MMP and TIMP levels warrant further investigation and may represent promising targets for future biomarker studies in sepsis. Nevertheless, extensive studies are required to validate these effects. We anticipate that these results will inform both future clinical and pharmacological research and assist subsequent studies.

## Figures and Tables

**Table 1:** MMP-1, MMP-2, MMP-3, MMP-8, MMP-9, MMP-13, TIMP-1, TIMP-2, TIMP-3, TNF-α, IL-6, IL-10, and IFN-γ levels were compared between Sham and LPS groups using in-dependent-group statistics and FDR correction. Mean, Standard error, *p*-value and FDR q-value are shown in the table.

Marker	Tissue	Sham Mean ± SE	LPS Mean ± SE	Test	*p*-Value	BH-FDR q
MMP-1	Serum	4.305 ± 0.741	9.032 ± 0.884	Independent-samples *t*-test	0.0023	0.0056
MMP-1	Brain	1.950 ± 0.417	3.615 ± 0.478	Mann–Whitney U	0.0152	0.0328
MMP-1	Kidney	2.743 ± 0.611	7.680 ± 0.879	Mann–Whitney U	0.0022	0.0056
MMP-2	Serum	4.355 ± 0.419	5.414 ± 0.795	Independent-samples *t*-test	0.2743	0.3821
MMP-2	Brain	3.426 ± 0.484	17.389 ± 1.920	Independent-samples *t*-test	0.0005	0.0034
MMP-2	Kidney	3.619 ± 0.502	4.870 ± 0.482	Independent-samples *t*-test	0.1025	0.1738
MMP-3	Serum	3.689 ± 0.282	18.700 ± 2.202	Independent-samples *t*-test	0.0009	0.0041
MMP-3	Brain	3.358 ± 0.620	5.039 ± 0.367	Independent-samples *t*-test	0.0474	0.0881
MMP-3	Kidney	3.361 ± 0.480	17.351 ± 1.652	Independent-samples *t*-test	0.0002	0.0030
MMP-8	Serum	8.859 ± 1.074	58.295 ± 7.063	Independent-samples *t*-test	0.0008	0.0039
MMP-8	Brain	8.360 ± 1.959	9.699 ± 1.696	Independent-samples *t*-test	0.6167	0.6625
MMP-8	Kidney	8.098 ± 1.831	10.250 ± 2.014	Mann–Whitney U	0.4848	0.5730
MMP-9	Serum	0.990 ± 0.130	3.537 ± 0.322	Independent-samples *t*-test	0.0002	0.0030
MMP-9	Brain	0.855 ± 0.093	1.069 ± 0.152	Independent-samples *t*-test	0.2617	0.3780
MMP-9	Kidney	0.969 ± 0.128	2.953 ± 0.300	Independent-samples *t*-test	0.0006	0.0034
MMP-13	Serum	1.845 ± 0.439	3.201 ± 0.478	Mann–Whitney U	0.0411	0.0802
MMP-13	Brain	2.145 ± 0.437	3.023 ± 0.422	Independent-samples *t*-test	0.1792	0.2795
MMP-13	Kidney	2.081 ± 0.344	9.353 ± 1.254	Independent-samples *t*-test	0.0016	0.0056
TIMP-1	Serum	0.859 ± 0.082	1.154 ± 0.174	Mann–Whitney U	0.3095	0.4024
TIMP-1	Brain	0.988 ± 0.102	0.864 ± 0.087	Independent-samples *t*-test	0.3778	0.4753
TIMP-1	Kidney	0.728 ± 0.070	3.092 ± 0.300	Independent-samples *t*-test	0.0004	0.0034
TIMP-2	Serum	4.816 ± 0.557	31.654 ± 3.762	Mann–Whitney U	0.0022	0.0056
TIMP-2	Brain	4.073 ± 0.512	25.086 ± 3.101	Mann–Whitney U	0.0022	0.0056
TIMP-2	Kidney	3.874 ± 0.585	3.546 ± 0.334	Independent-samples *t*-test	0.6393	0.6625
TIMP-3	Serum	0.887 ± 0.093	0.978 ± 0.117	Independent-samples *t*-test	0.5550	0.6366
TIMP-3	Brain	0.852 ± 0.096	0.829 ± 0.148	Mann–Whitney U	0.4848	0.5730
TIMP-3	Kidney	0.813 ± 0.103	4.487 ± 0.721	Mann–Whitney U	0.0022	0.0056
TNF-α	Serum	29.726 ± 6.547	25.605 ± 5.284	Mann–Whitney U	0.8182	0.8182
TNF-α	Brain	27.253 ± 7.348	23.194 ± 4.262	Independent-samples *t*-test	0.6455	0.6625
TNF-α	Kidney	26.376 ± 6.278	35.882 ± 5.839	Independent-samples *t*-test	0.2936	0.3949
IL-6	Serum	4.162 ± 0.418	10.511 ± 1.596	Mann–Whitney U	0.0022	0.0056
IL-6	Brain	3.693 ± 0.520	4.044 ± 0.586	Mann–Whitney U	0.5887	0.6560
IL-6	Kidney	3.530 ± 0.568	4.946 ± 0.755	Independent-samples *t*-test	0.1671	0.2715
IL-10	Serum	26.672 ± 2.385	242.673 ± 23.373	Independent-samples *t*-test	0.0002	0.0030
IL-10	Brain	23.810 ± 2.547	34.207 ± 2.366	Independent-samples *t*-test	0.0136	0.0313
IL-10	Kidney	23.626 ± 4.434	30.486 ± 2.167	Mann–Whitney U	0.2403	0.3604
IFN-γ	Serum	10.416 ± 2.518	15.318 ± 0.996	Mann–Whitney U	0.0649	0.1151
IFN-γ	Brain	19.029 ± 3.000	110.616 ± 12.818	Independent-samples *t*-test	0.0006	0.0034
IFN-γ	Kidney	13.083 ± 2.696	23.368 ± 2.158	Mann–Whitney U	0.0260	0.0533

## Data Availability

The original contributions presented in this study are included in the article. Further inquiries can be directed to the corresponding author.

## Abbreviations

The following abbreviations are used in this manuscript:

ECM	Extracellular matrix
MMP	Matrix metalloproteinases
TIMP	Tissue inhibitors of matrix metalloproteinases
IFN-γ	Interferon gamma
IL-6	Interleukin-6
IL-10	Interleukin-10
TNF-α	Tumor Necrosis Factor alpha
